# Supplementary material for: Introduction of a leaky stop codon as molecular tool in Chlamydomonas reinhardtii
Source: PLoS One. 2020 Aug 20;15(8):e0237405. doi: 10.1371/journal.pone.0237405 (PMC7440625; doi:10.1371/journal.pone.0237405)

Fig 1C

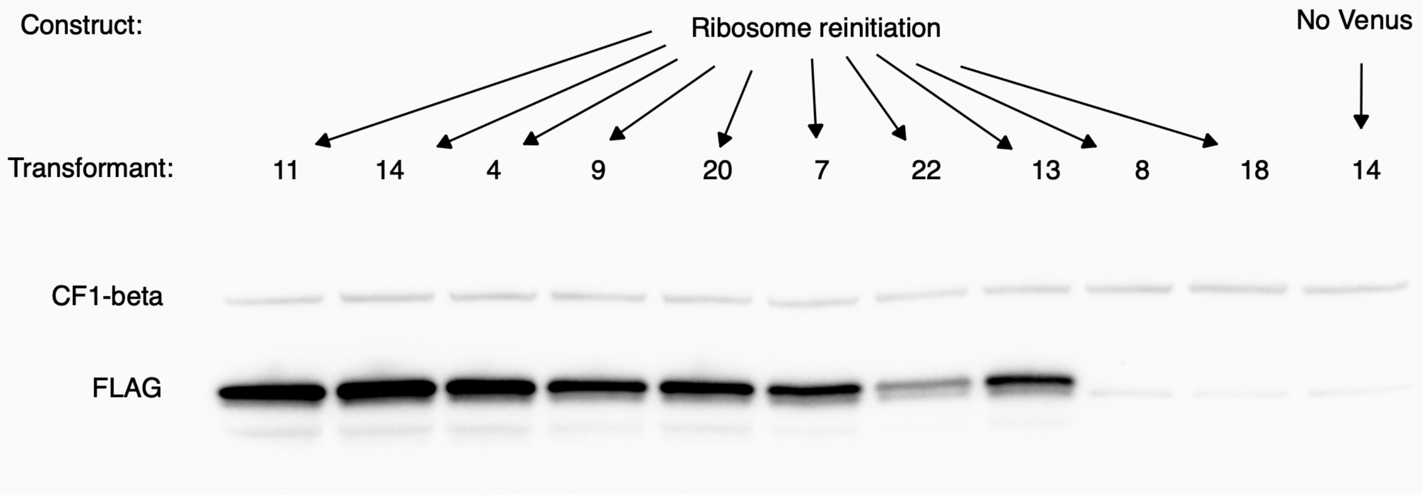

Construct:
Ribosome reinitiation
No Venus
Transformant:
11
14
4
9
20
7
22
13
8
18
14
CF1-beta
FLAG

Fig 1C - corresponding membrane

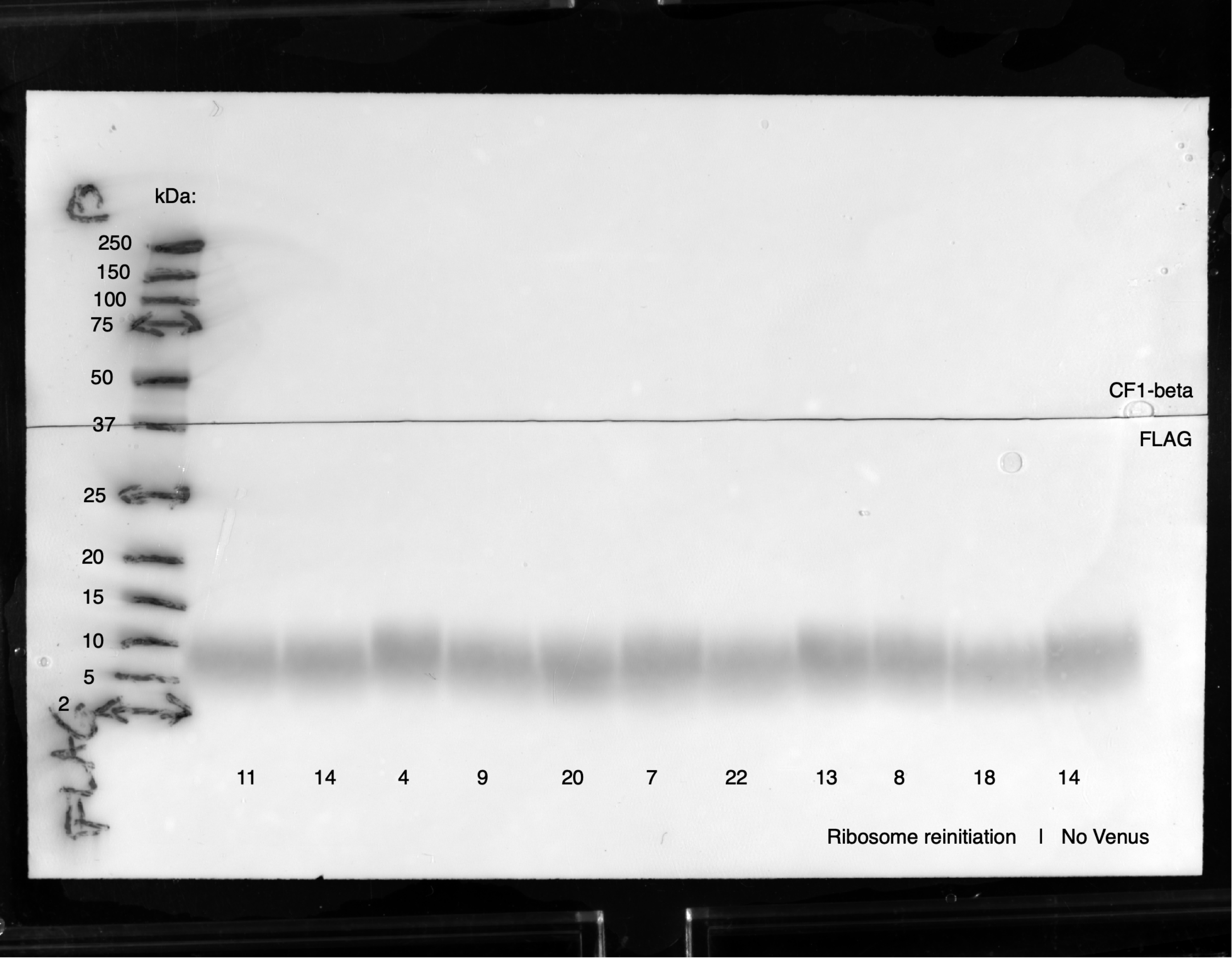

Fig 3A - Rbc

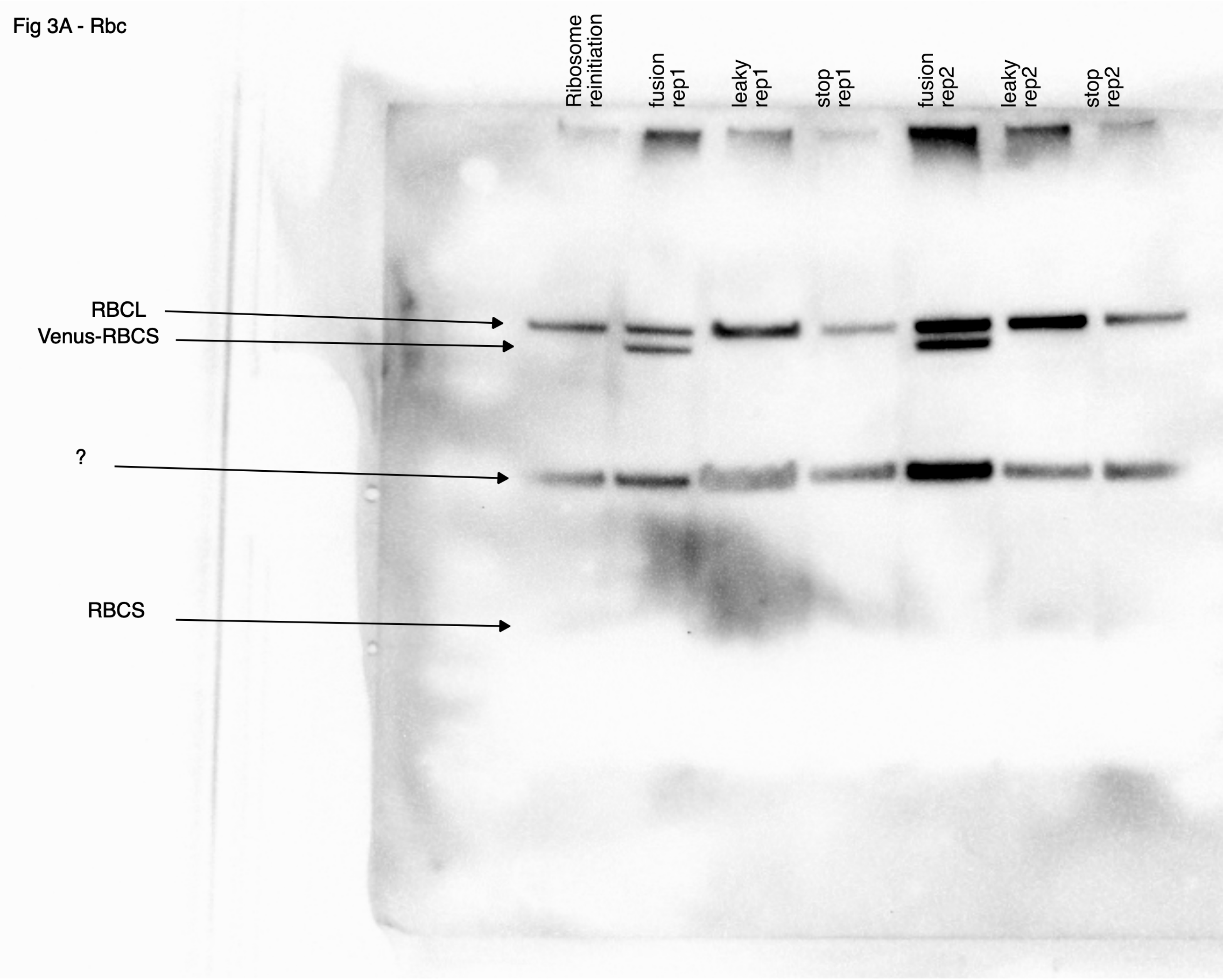
Ribosome reinitiation
fusion rep1
leaky rep1
stop rep1
fusion rep2
leaky rep2
stop rep2
RBCL
Venus-RBCS
?
RBCS

Fig 3A - Rbc - corresponding membrane

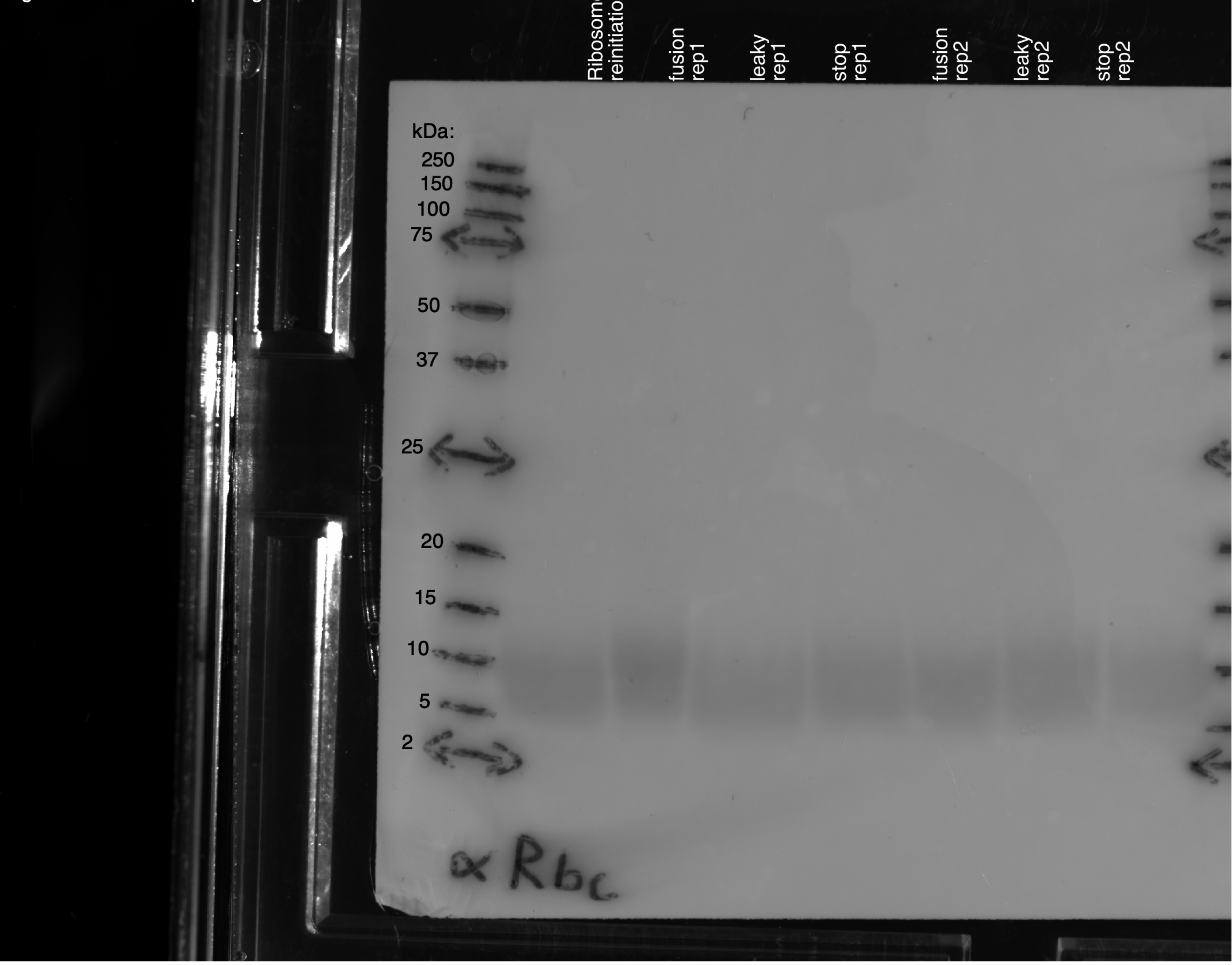

Fig 3A - FLAG

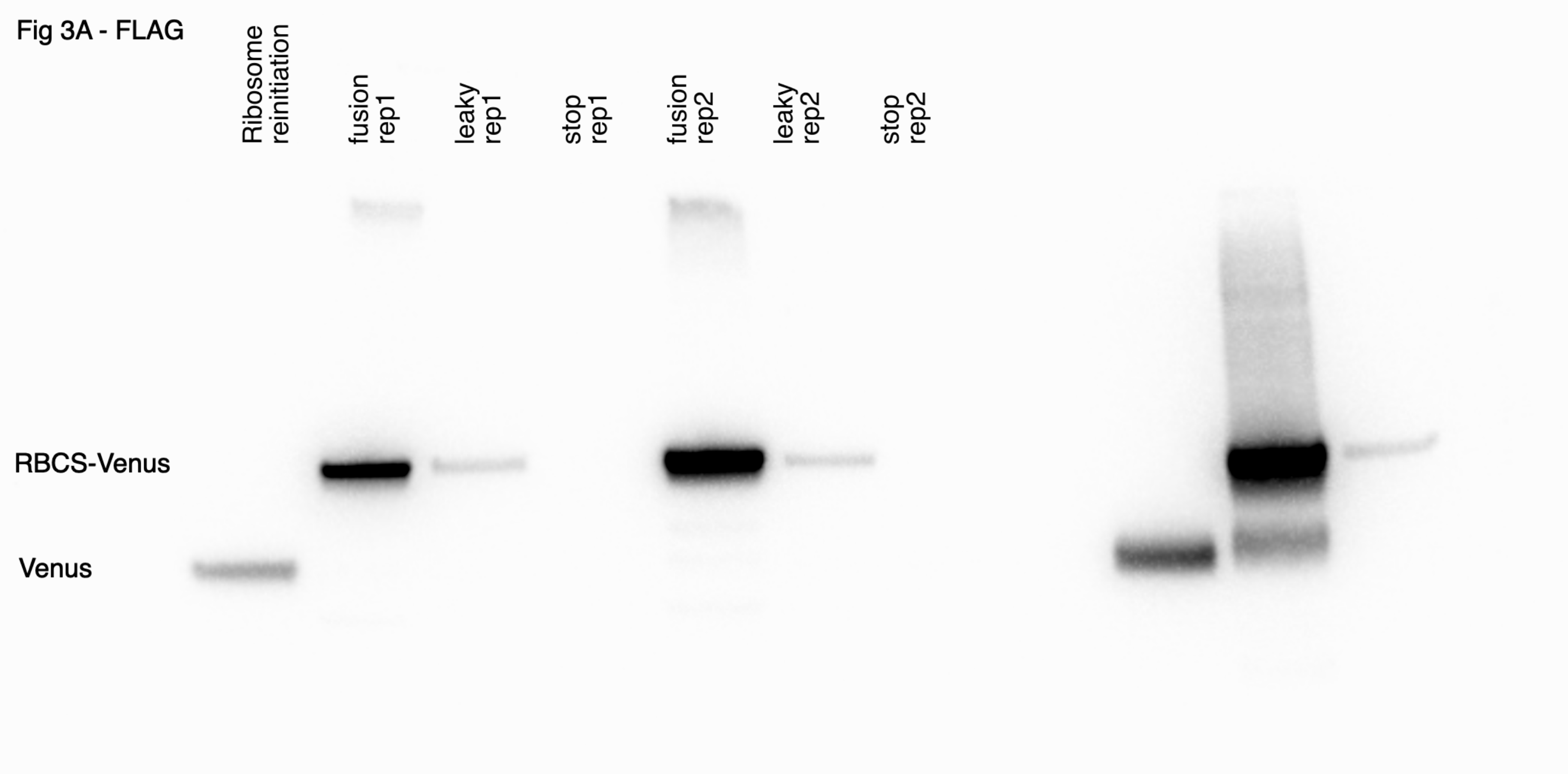
Ribosome reinitiation
fusion rep1
leaky rep1
stop rep1
fusion rep2
leaky rep2
stop rep2
RBCS-Venus
Venus

Fig 3A - FLAG - corresponding membrane

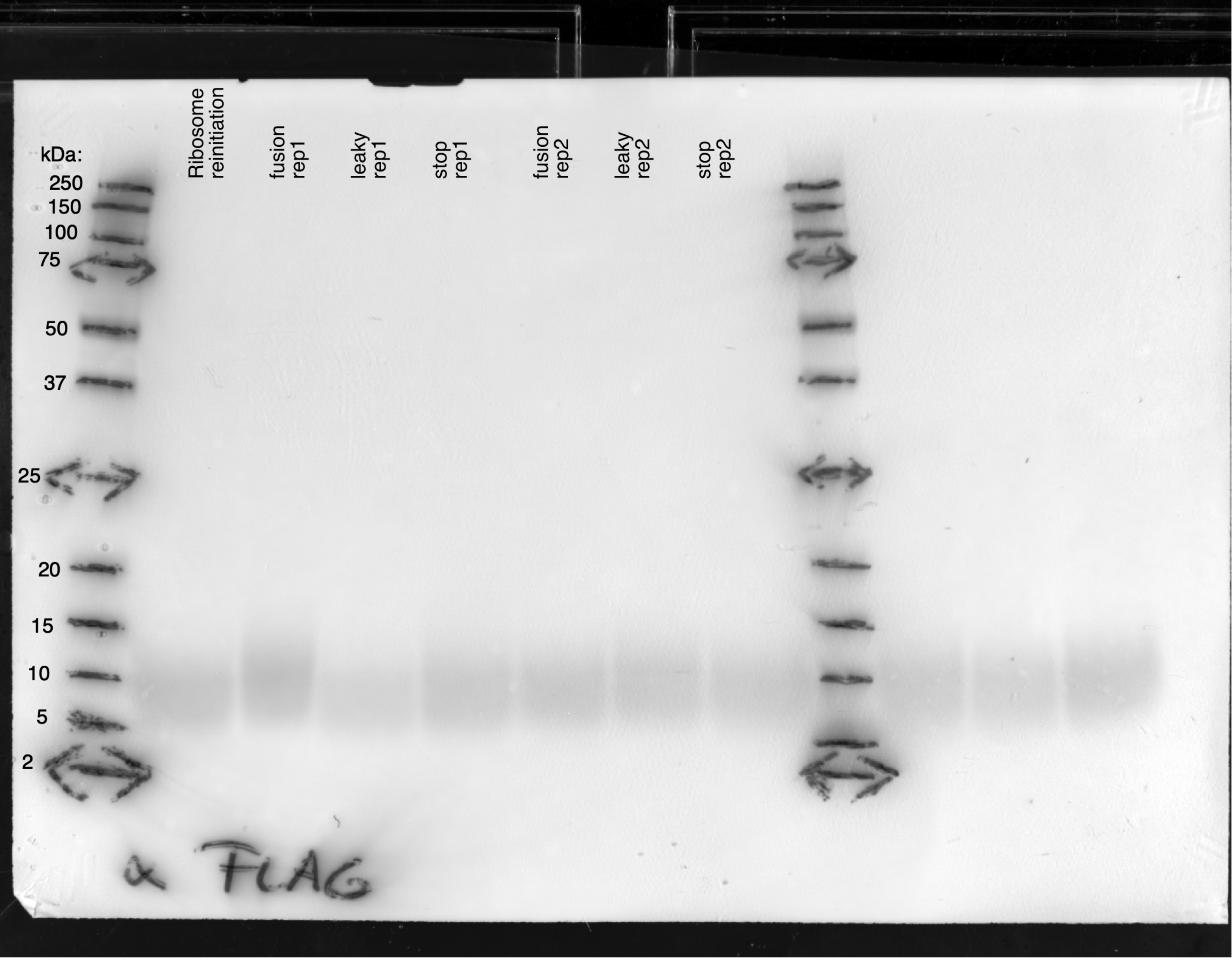

Supplement: S1 Raw images — (PDF) [file pone.0237405.s001.pdf]
