# Supplementary figures and images for: Introduction of a leaky stop codon as molecular tool in Chlamydomonas reinhardtii
Source: PLoS One. 2020 Aug 20;15(8):e0237405. doi: 10.1371/journal.pone.0237405 (PMC7440625; doi:10.1371/journal.pone.0237405)

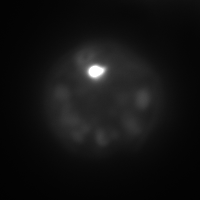

Supplement: S2 Data — (ZIP) [file pone.0237405.s003.zip › S3_microscopy_original/T60 - fusion (pODC20)/20180430_c:1:3 - T-60_pODC20_06-04.czi #1.tif]

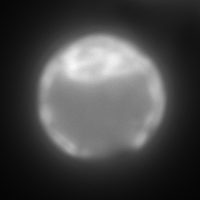

Supplement: S2 Data — (ZIP) [file pone.0237405.s003.zip › S3_microscopy_original/T60 - fusion (pODC20)/20180430_c:2:3 - T-60_pODC20_06-04.czi #1.tif]

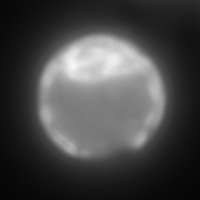

Supplement: S2 Data — (ZIP) [file pone.0237405.s003.zip › S3_microscopy_original/T60 - fusion (pODC20)/20180430_c:1+2:3 - T-60_pODC20_06-04.czi #1.tif]

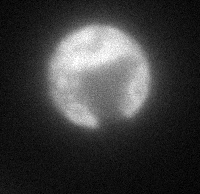

Supplement: S2 Data — (ZIP) [file pone.0237405.s003.zip › S3_microscopy_original/T60 - stop (pODC22)/20180430_c:1:3 - T-60_pODC22_07.czi #1.tif]

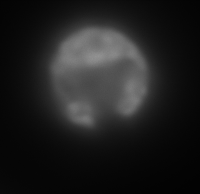

Supplement: S2 Data — (ZIP) [file pone.0237405.s003.zip › S3_microscopy_original/T60 - stop (pODC22)/20180430_c:1+2:3 - T-60_pODC22_07.czi #1.tif]

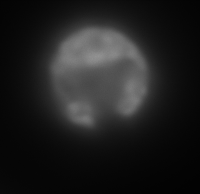

Supplement: S2 Data — (ZIP) [file pone.0237405.s003.zip › S3_microscopy_original/T60 - stop (pODC22)/20180430_c:2:3 - T-60_pODC22_07.czi #1.tif]

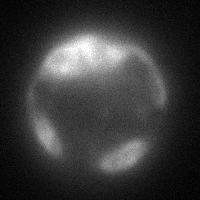

Supplement: S2 Data — (ZIP) [file pone.0237405.s003.zip › S3_microscopy_original/Cal13.1B - stop (pODC22)/20180430_c:1:3 - Cal13.1B_pODC22_07-06.czi #1.tif]

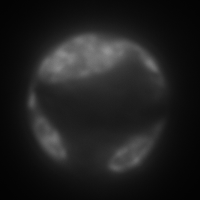

Supplement: S2 Data — (ZIP) [file pone.0237405.s003.zip › S3_microscopy_original/Cal13.1B - stop (pODC22)/20180430_c:1+2:3 - Cal13.1B_pODC22_07-06.czi #1.tif]

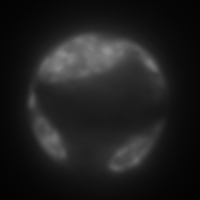

Supplement: S2 Data — (ZIP) [file pone.0237405.s003.zip › S3_microscopy_original/Cal13.1B - stop (pODC22)/20180430_c:2:3 - Cal13.1B_pODC22_07-06.czi #1.tif]

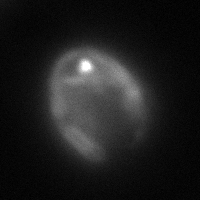

Supplement: S2 Data — (ZIP) [file pone.0237405.s003.zip › S3_microscopy_original/Cal13.1B - leaky (pODC21)/20180430_c:1:3 - Cal13.1B_pODC21_03-08.czi #1.tif]

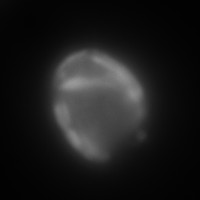

Supplement: S2 Data — (ZIP) [file pone.0237405.s003.zip › S3_microscopy_original/Cal13.1B - leaky (pODC21)/20180430_c:1+2:3 - Cal13.1B_pODC21_03-08.czi #1.tif]

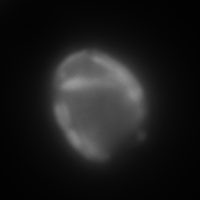

Supplement: S2 Data — (ZIP) [file pone.0237405.s003.zip › S3_microscopy_original/Cal13.1B - leaky (pODC21)/20180430_c:2:3 - Cal13.1B_pODC21_03-08.czi #1.tif]

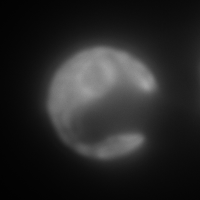

Supplement: S2 Data — (ZIP) [file pone.0237405.s003.zip › S3_microscopy_original/T60 - leaky (pODC21)/20180430_c:1+2:3 - T-60_pODC21_08.czi #1.tif]

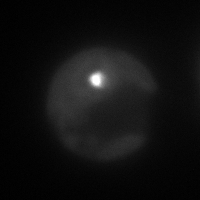

Supplement: S2 Data — (ZIP) [file pone.0237405.s003.zip › S3_microscopy_original/T60 - leaky (pODC21)/20180430_c:1:3 - T-60_pODC21_08.czi #1.tif]

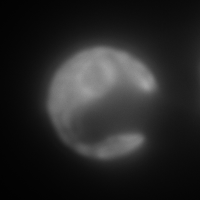

Supplement: S2 Data — (ZIP) [file pone.0237405.s003.zip › S3_microscopy_original/T60 - leaky (pODC21)/20180430_c:2:3 - T-60_pODC21_08.czi #1.tif]

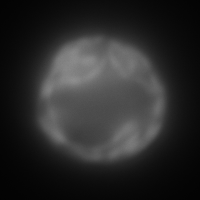

Supplement: S2 Data — (ZIP) [file pone.0237405.s003.zip › S3_microscopy_original/Cal13.1B - fusion (pODC20)/20180430_c:1+2:3 - Cal13.1B_pODC20_17.czi #1.tif]

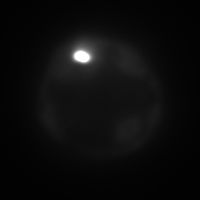

Supplement: S2 Data — (ZIP) [file pone.0237405.s003.zip › S3_microscopy_original/Cal13.1B - fusion (pODC20)/20180430_c:1:3 - Cal13.1B_pODC20_17.czi #1.tif]

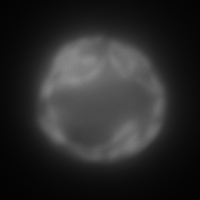

Supplement: S2 Data — (ZIP) [file pone.0237405.s003.zip › S3_microscopy_original/Cal13.1B - fusion (pODC20)/20180430_c:2:3 - Cal13.1B_pODC20_17.czi #1.tif]

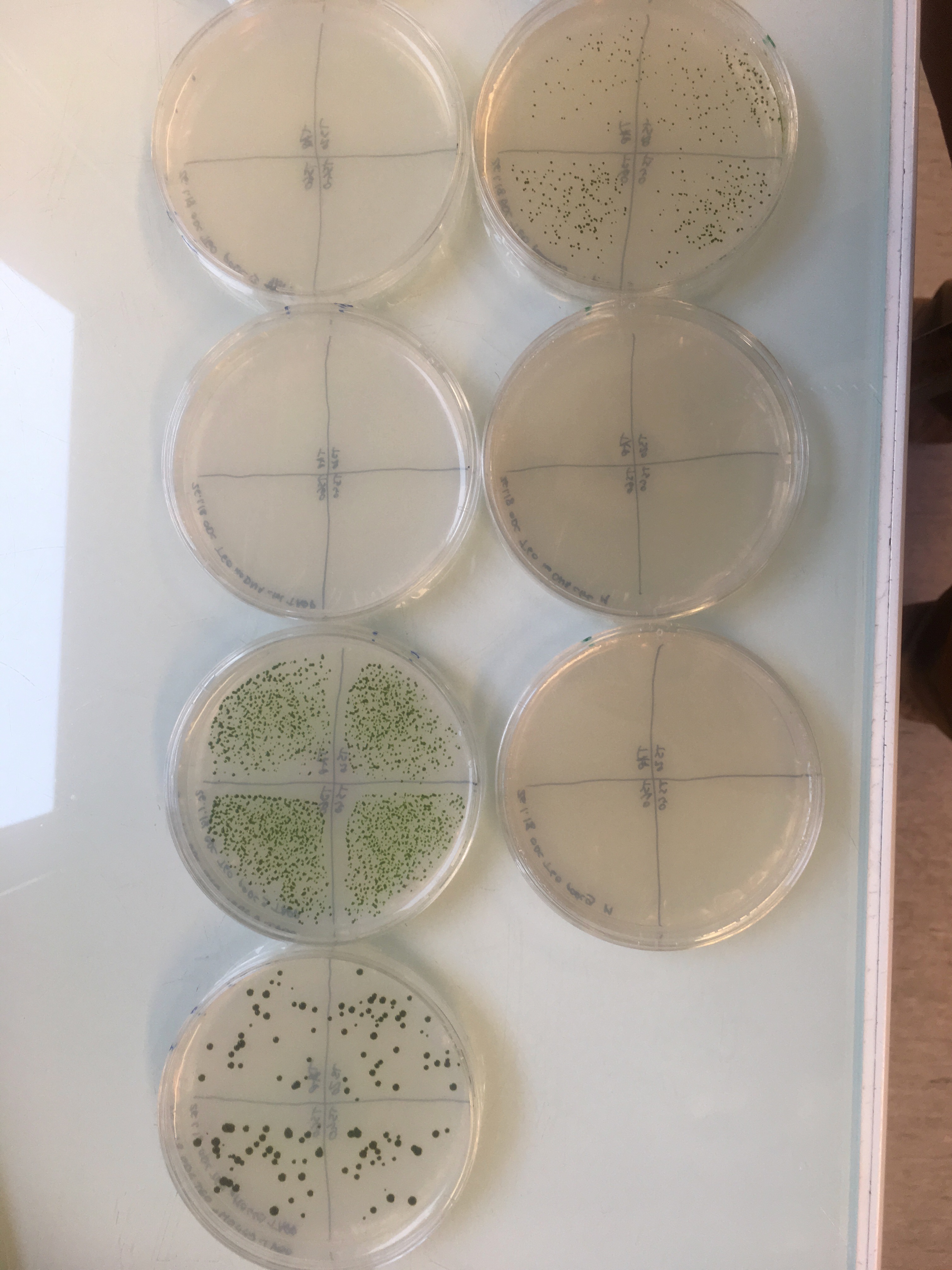

Supplement: S3 Data — (ZIP) [file pone.0237405.s004.zip › T60 rep1/T60_control_top.jpeg]

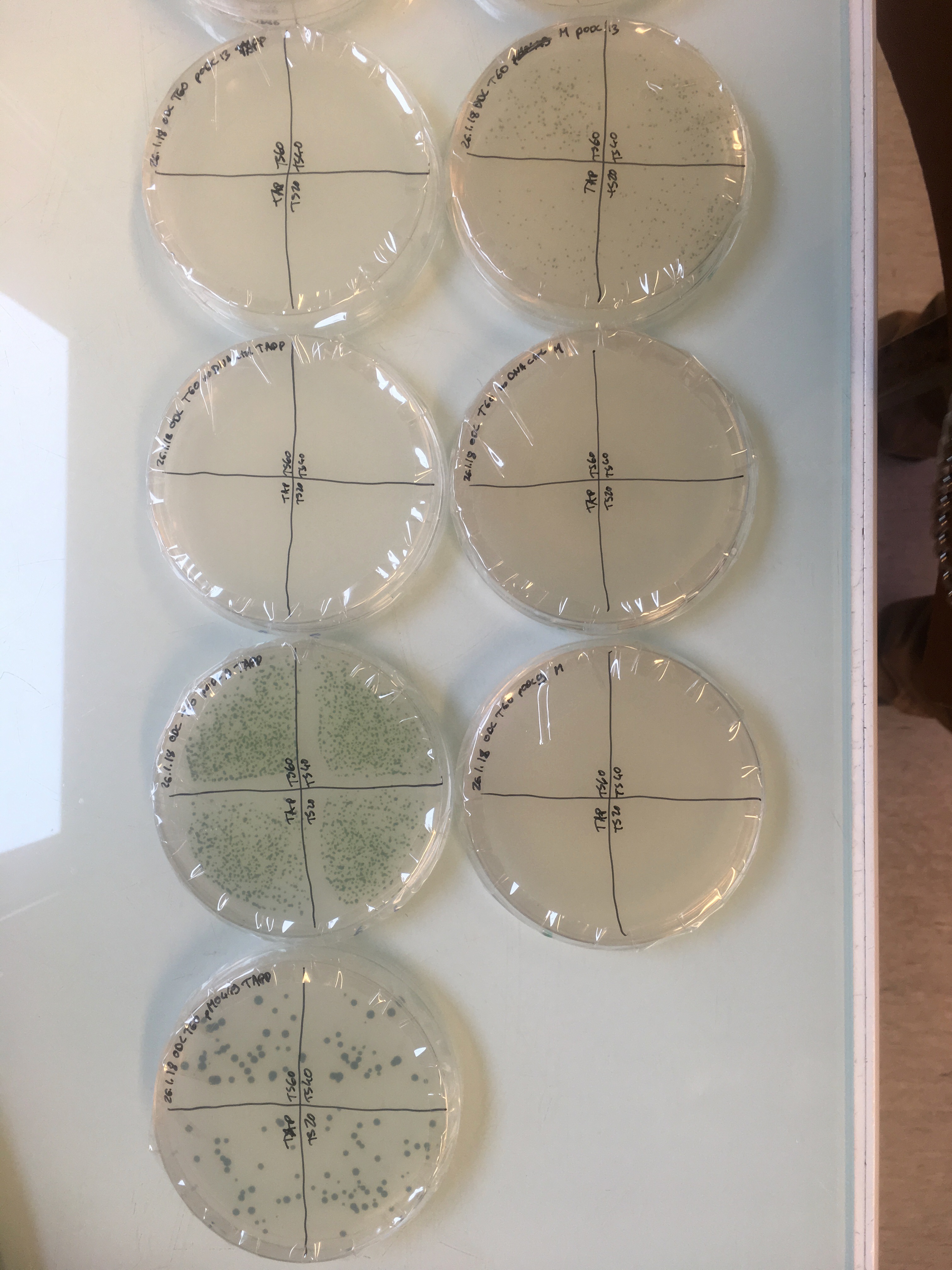

Supplement: S3 Data — (ZIP) [file pone.0237405.s004.zip › T60 rep1/T-60_controls_bottom.jpeg]

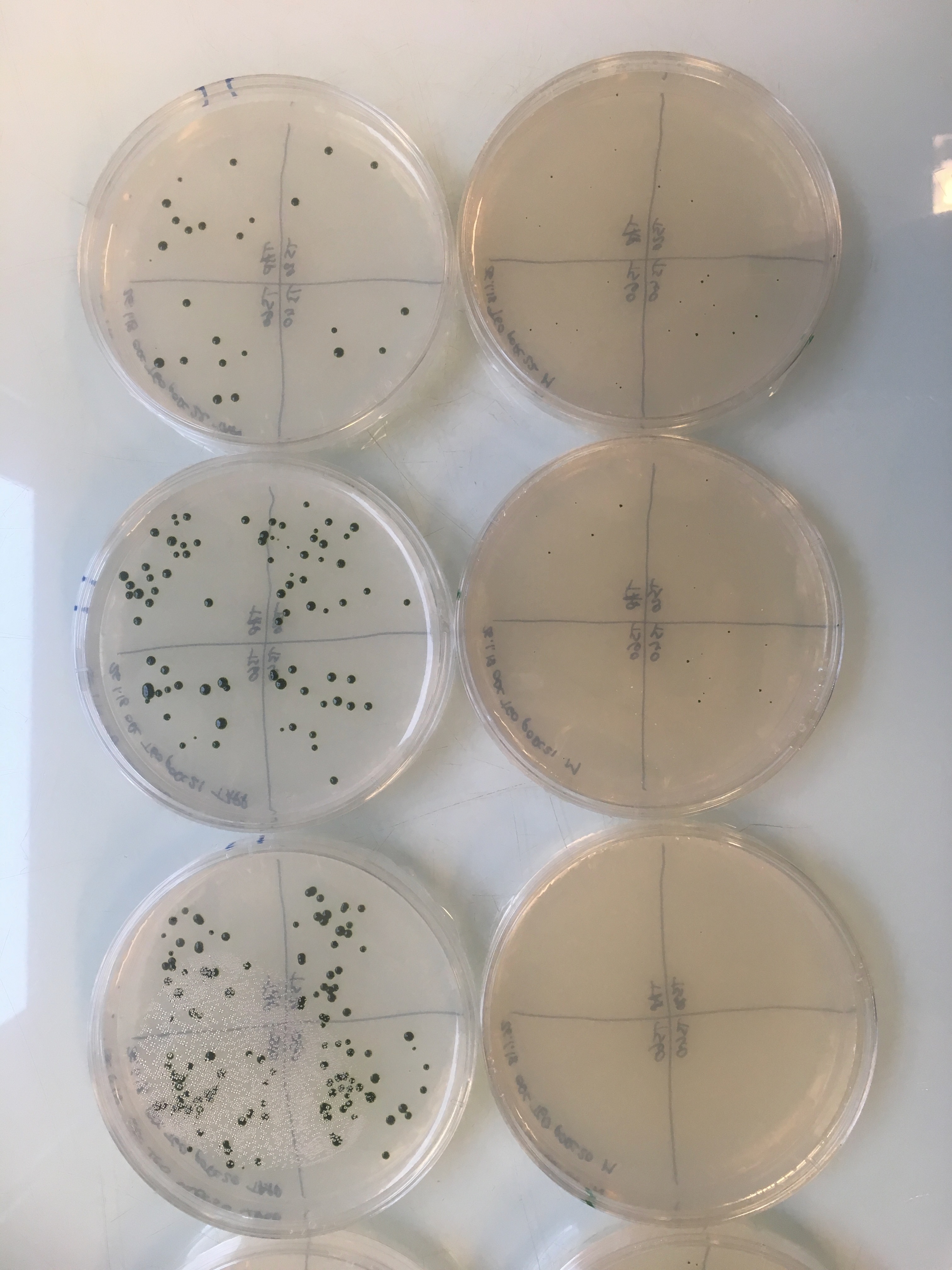

Supplement: S3 Data — (ZIP) [file pone.0237405.s004.zip › T60 rep1/T60_samples_top.jpeg]

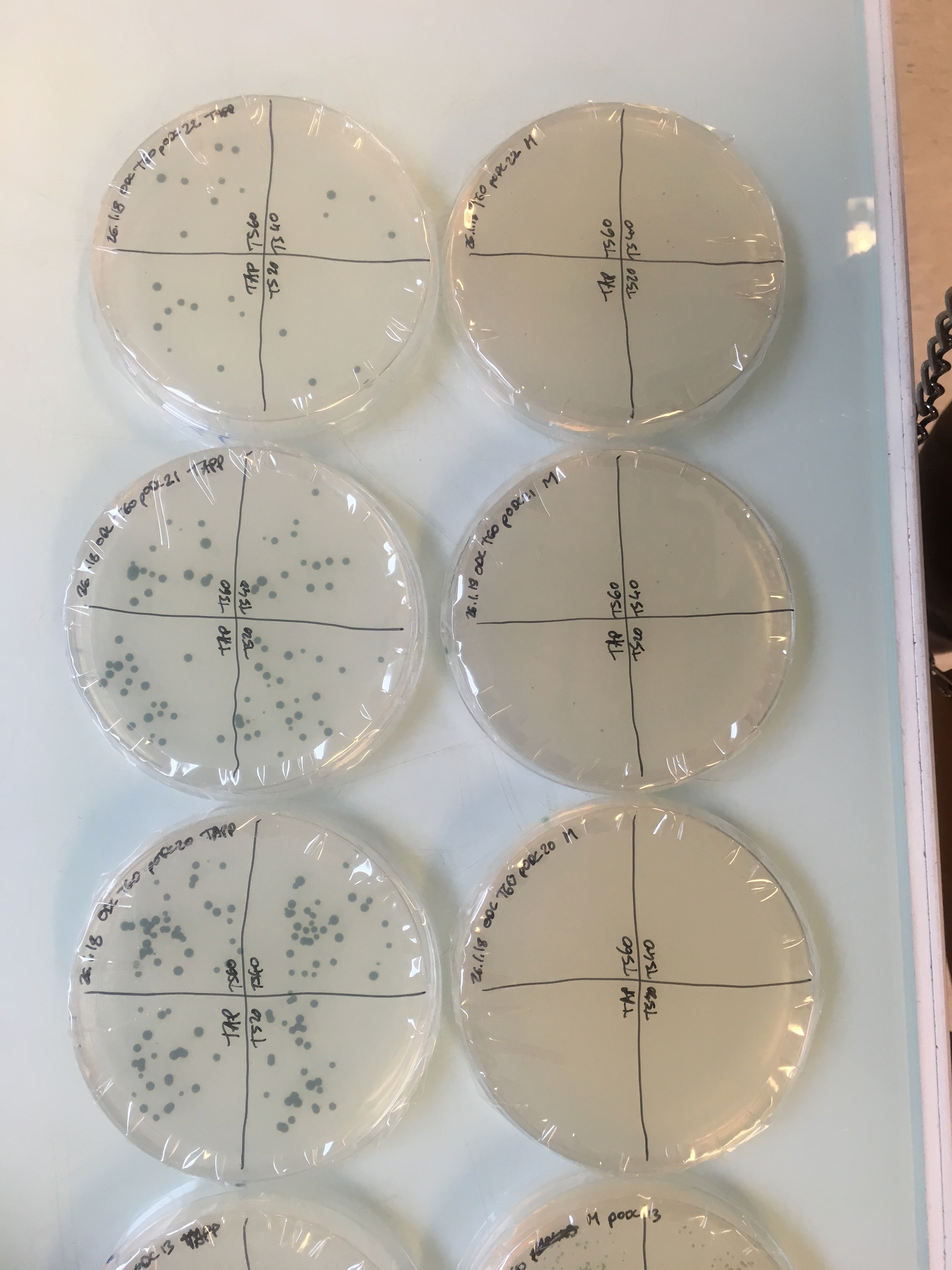

Supplement: S3 Data — (ZIP) [file pone.0237405.s004.zip › T60 rep1/T-60_samples_bottom.jpeg]

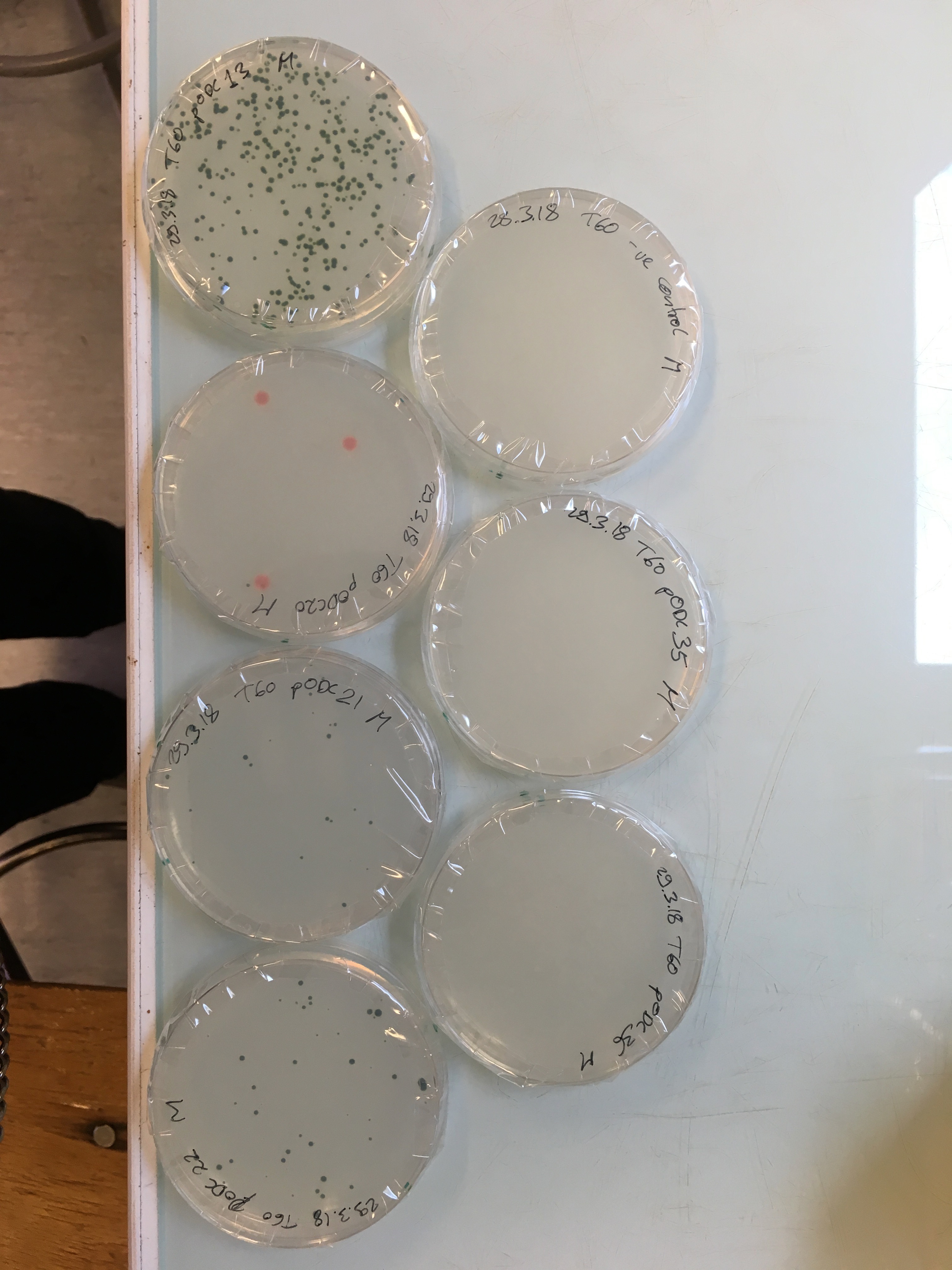

Supplement: S4 Data — (ZIP) [file pone.0237405.s005.zip › T60 rep2/T60_M_bottom.jpeg]

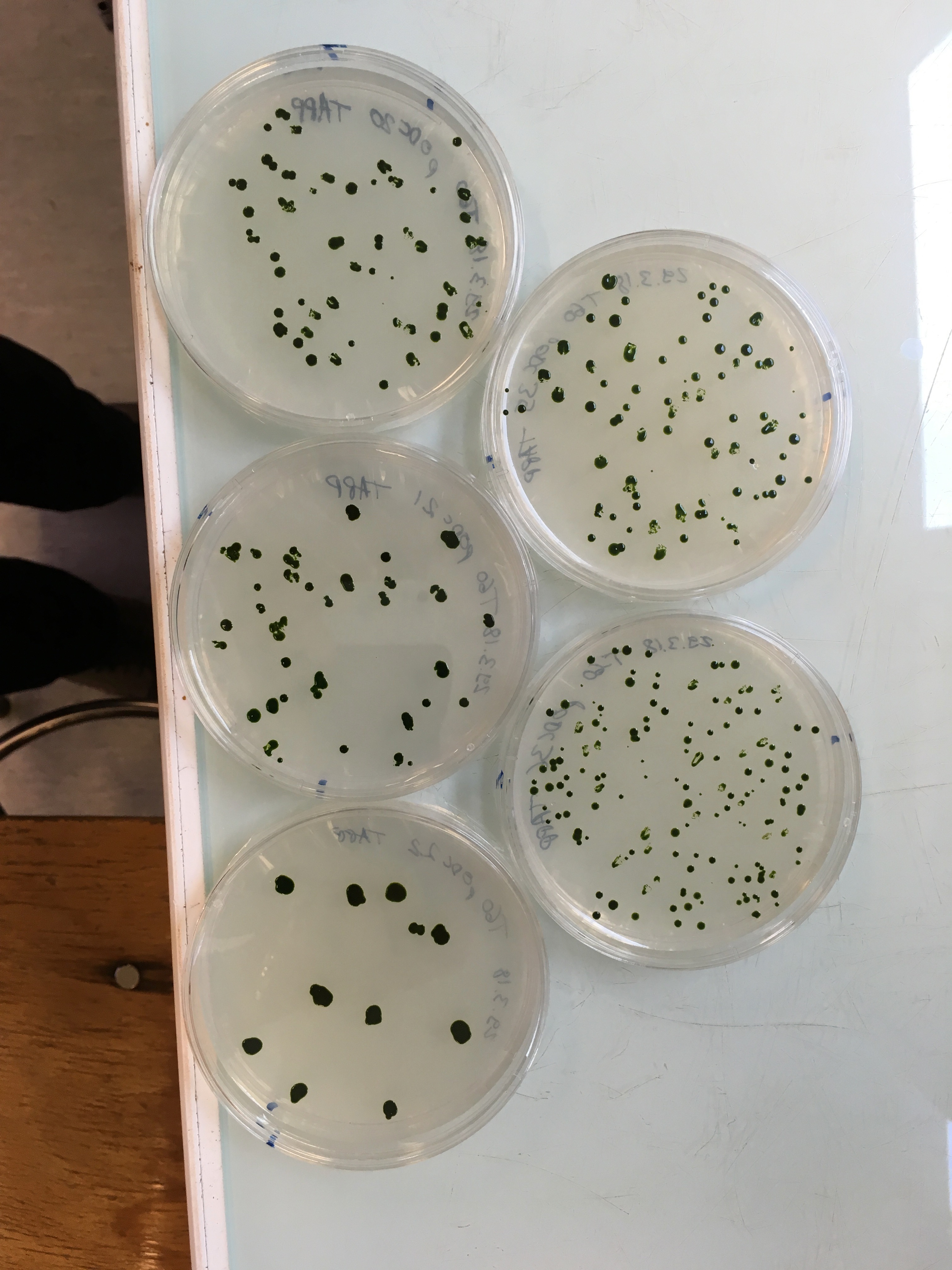

Supplement: S4 Data — (ZIP) [file pone.0237405.s005.zip › T60 rep2/T60_TAPP_top.jpeg]

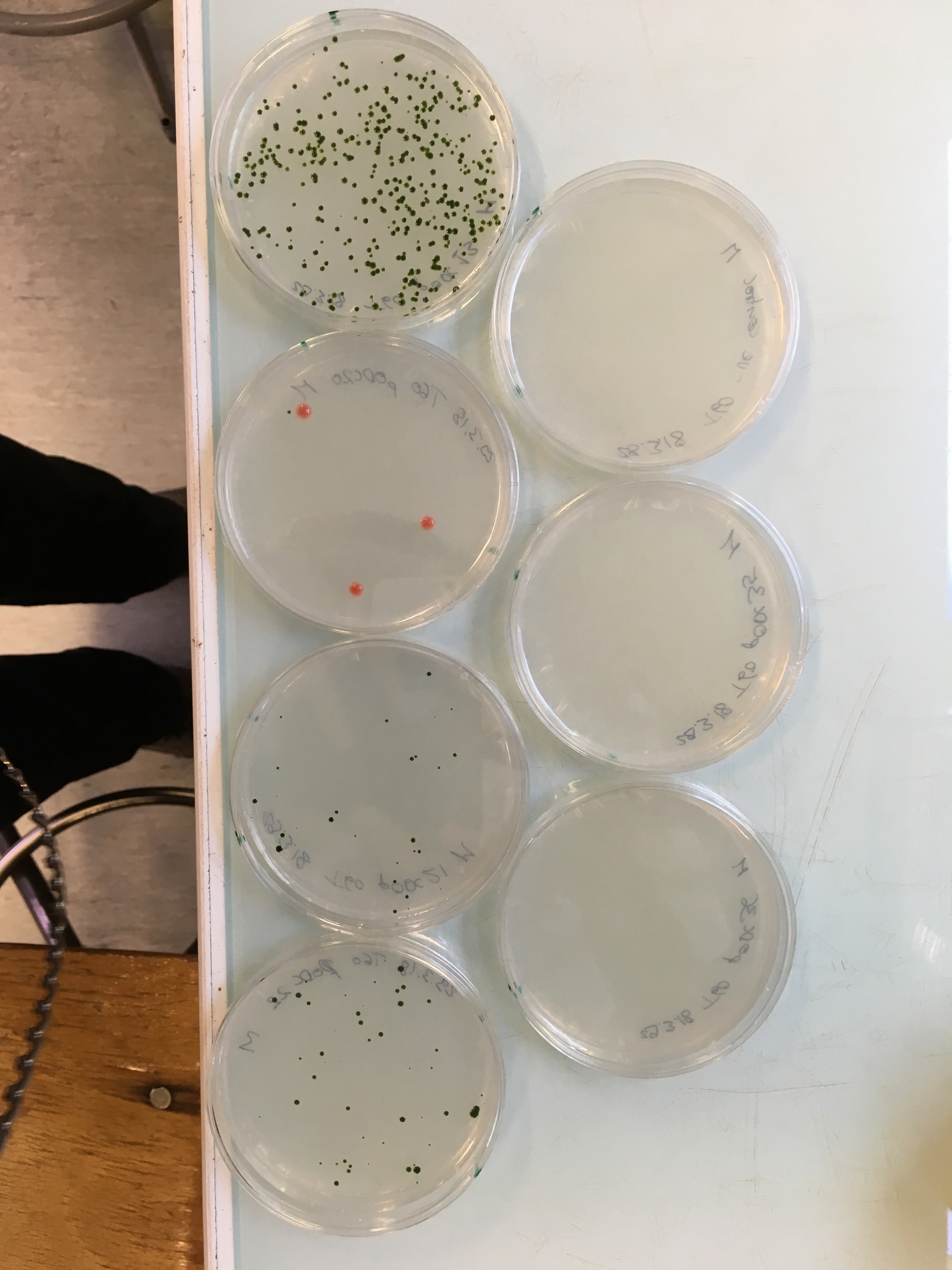

Supplement: S4 Data — (ZIP) [file pone.0237405.s005.zip › T60 rep2/T-60_M_top.jpeg]

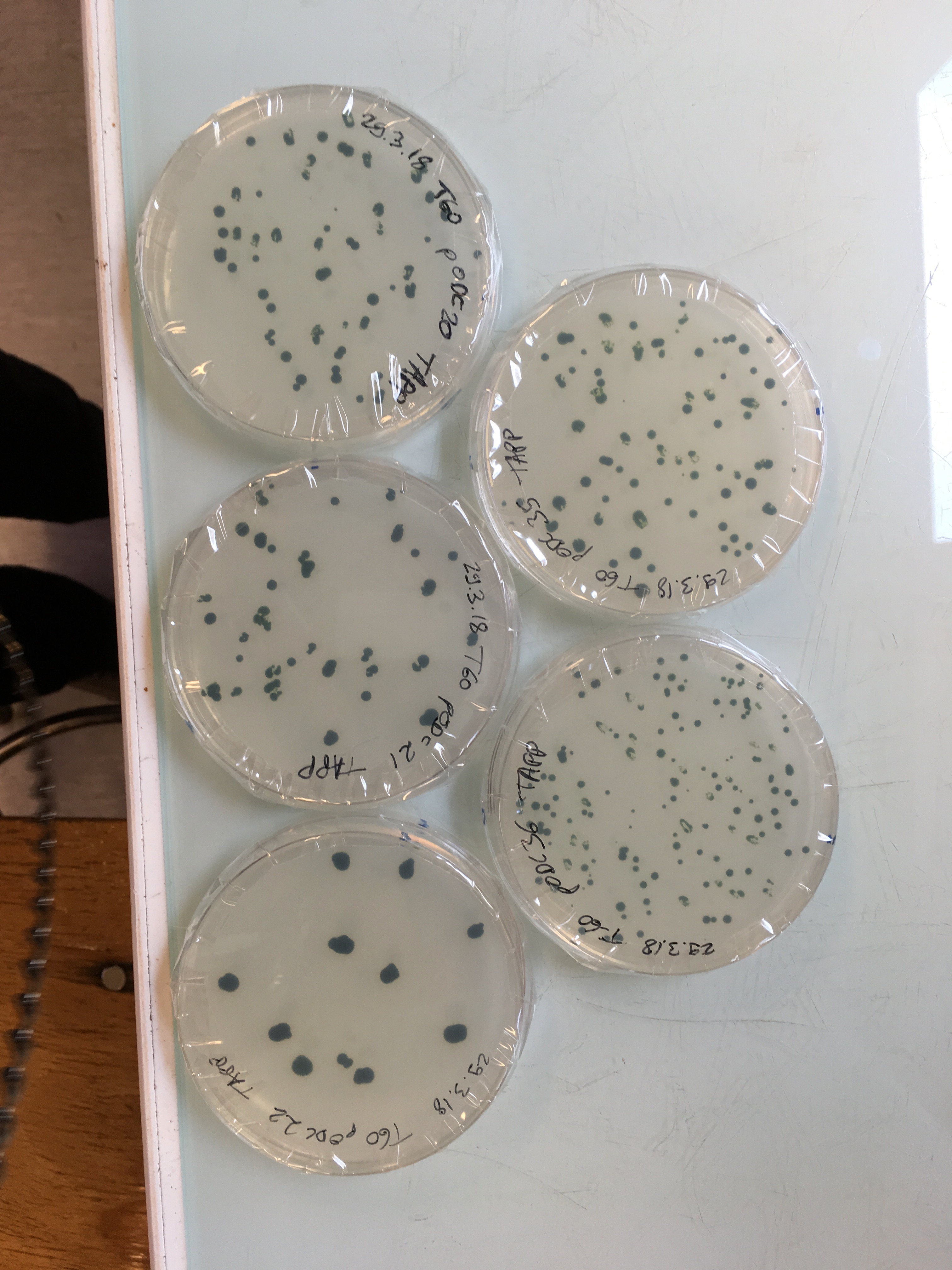

Supplement: S4 Data — (ZIP) [file pone.0237405.s005.zip › T60 rep2/T60_TAPP_bottom.jpeg]

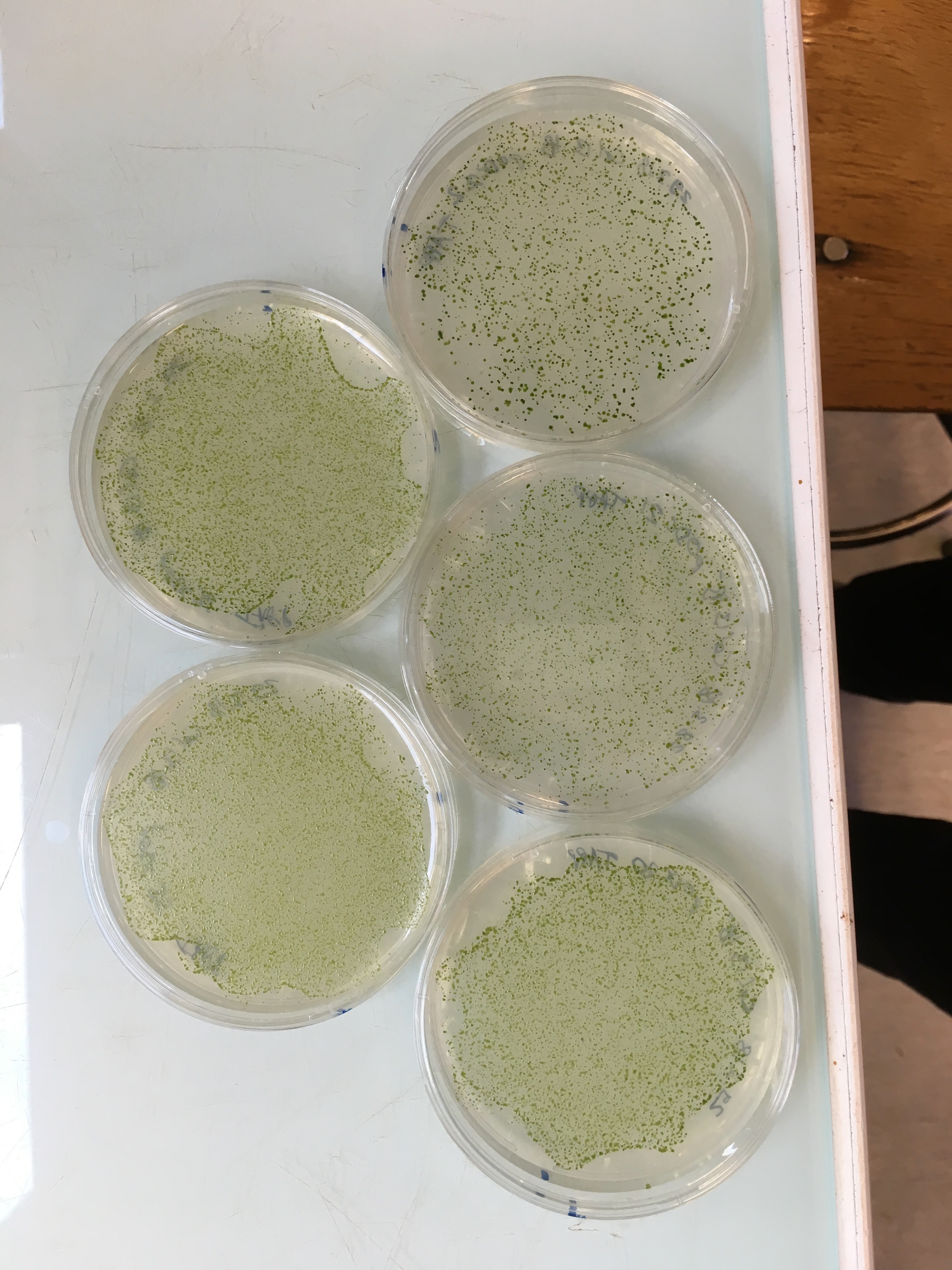

Supplement: S5 Data — (ZIP) [file pone.0237405.s006.zip › Cal13.1B-/Cal13.1B_TAPP_top.jpeg]

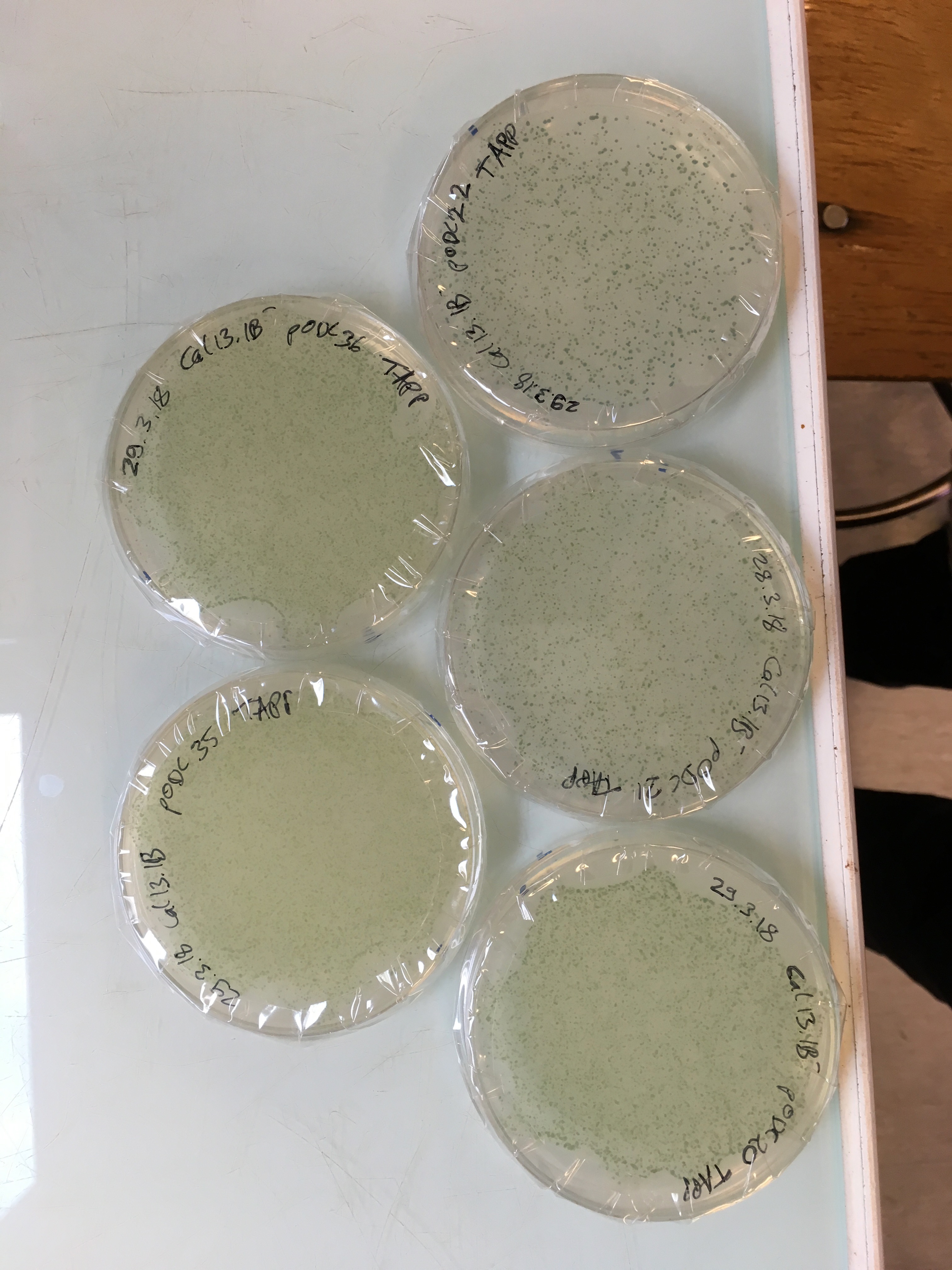

Supplement: S5 Data — (ZIP) [file pone.0237405.s006.zip › Cal13.1B-/Cal13.1B_TAPP_bottom.jpeg]

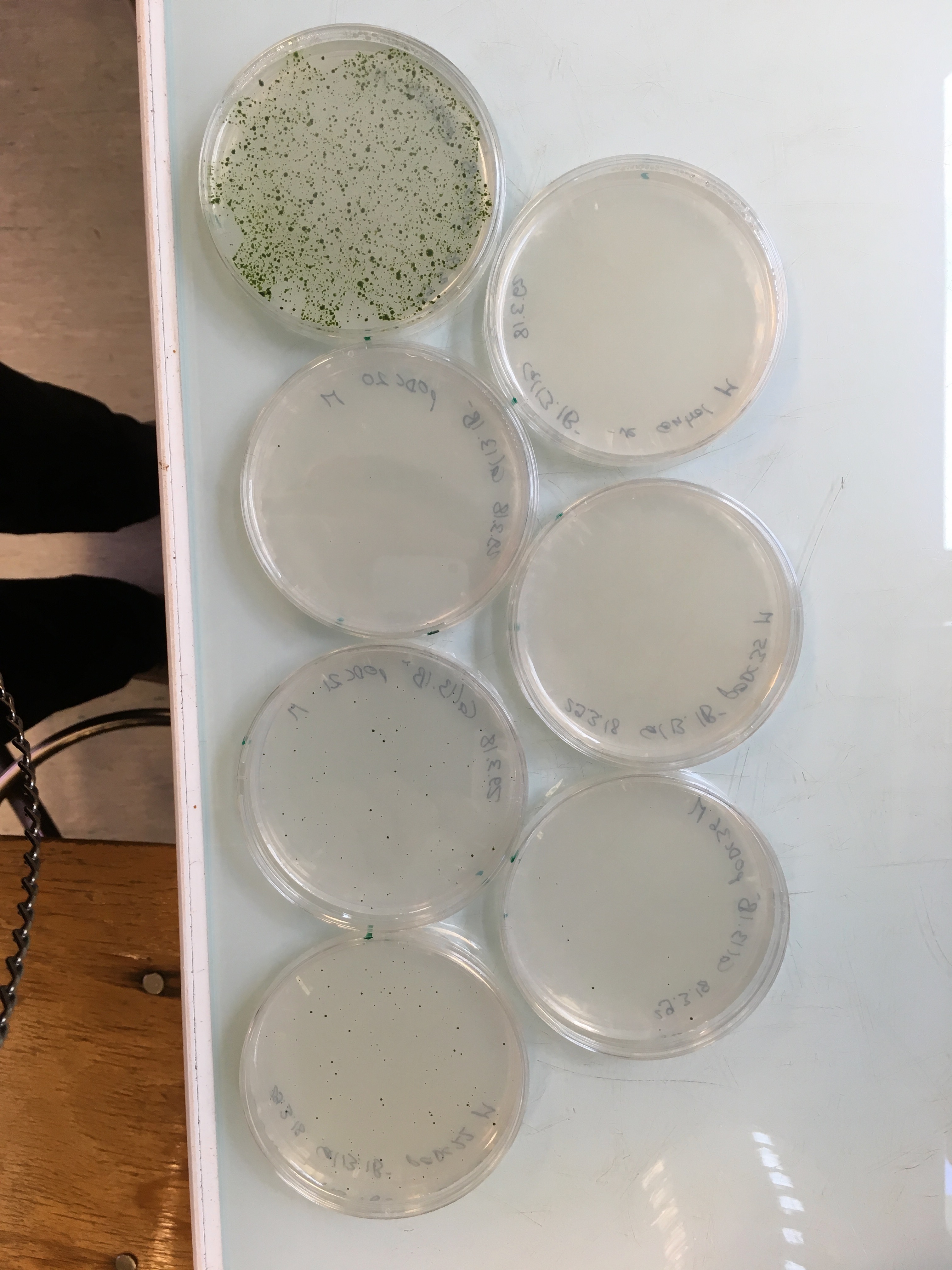

Supplement: S5 Data — (ZIP) [file pone.0237405.s006.zip › Cal13.1B-/Cal13.1B_M_top.jpeg]

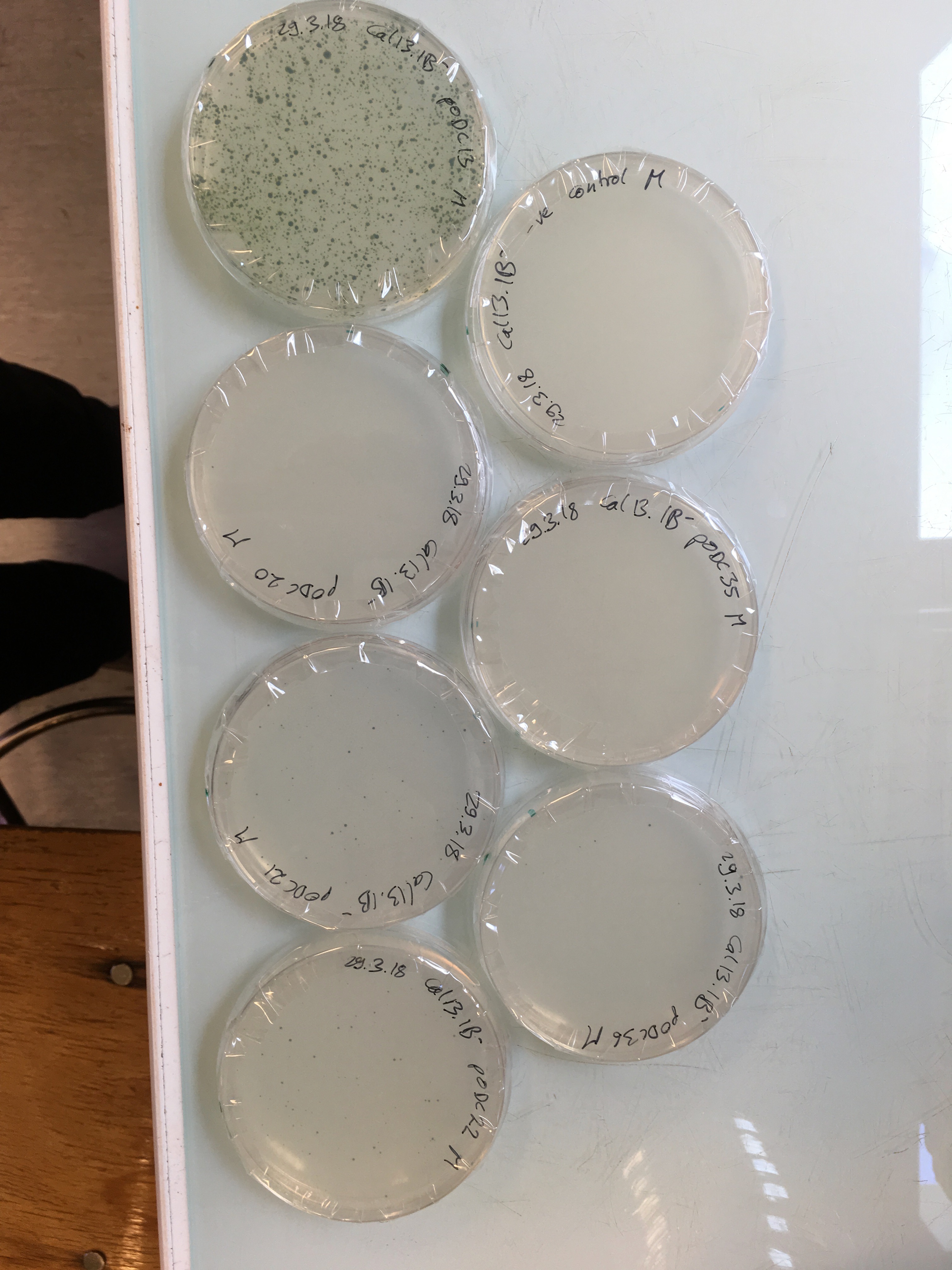

Supplement: S5 Data — (ZIP) [file pone.0237405.s006.zip › Cal13.1B-/Cal13.1B_M_bottom.jpeg]
