# Supplementary material for: Introduction of a leaky stop codon as molecular tool in Chlamydomonas reinhardtii
Source: PLoS One. 2020 Aug 20;15(8):e0237405. doi: 10.1371/journal.pone.0237405 (PMC7440625; doi:10.1371/journal.pone.0237405)
Supplement: Attachment — Submitted filename: Response to Reviewers.docx [file pone.0237405.s007.docx]

# Response to Reviewers

I thank David L Herrin for his helpful public review and am glad to hear he thinks people in the community will find my work useful.

Point 1: (Figure 1D and corresponding legend and text)

I agree with him that protein abundances should not be compared directly when inferred from different antibodies without absolute quantification. Most certainly I would expect there to be more CF1 beta than Venus. My use of CF1 beta in Fig. 1D was indeed as a loading control, and I simply used the resulting values directly as an arbitrary unit scale, if you will, to plot Venus quantities on. However, I must admit that this can very easily be misunderstood, and I have thus changed the scale to units relative to the average across my Venus samples. As I did refer to quantities of Venus relative to the population in the text anyway (**lines 185-190**), this should make the paper easier to understand. Supplementary Table S2 has been updated accordingly.

Indeed the reader should not need Ouija boards or tarot cards, I apologize for the oversight of not having indicated loaded quantities. Crude protein extracts were normalized to 2µg chlorophyll in each lane, which I have now specified in figure legends (**lines 158, 271**). A description of how bands were revealed was added in the methods section (**lines 431-434**).

An absolute quantification of Venus would certainly strengthen the case for saying a lot of protein is needed to generate detectable fluorescence. However, I do think the relative quantification I present is suggestive enough in its own right to state the observation. I have weakened the statement in the text to reflect the ambiguity that remains in the absence of absolute quantification (**lines 183-190, line 325**).

Point 2: (Figure 3A and corresponding legend and text)

Again apologies for the oversight, I have now added a clear statement about the identity of the control lane in the legend (**lines 273-274**); it is a ‘ribosome reinitiation’ construct transformant.

I understand that the relative quantification of Bands is confusing in that none of the lanes correspond to 100%. However, upon reflection I have decided to keep it that way. This is because 100% is meant to be the ‘real’ protein abundance in ‘fusion’ construct transformants. In the figure, I show two different ‘fusion’ construct transformants, which vary a lot in Venus abundance. If I were to set either of the two replicates to 100%, then I would get an arbitrarily high or low apparent abundance for the ‘leaky’ construct transformants. I do not think it makes sense to treat ‘leaky’ rep1 relative to ‘fusion’ rep1 and ‘leaky’ rep2 relative to ‘fusion’ rep2 either, since there is no *a priori* reason why they should form sets. Rather, differences in expression levels should be down to differences in the genomic locus that the transgene construct has integrated in. Thus the best thing to do, in my mind, is to take the average of the two ‘fusion’ transformants and set that value to 100%. This is what I have done, and I have updated the legend in a way that hopefully makes this clearer (**lines 275-276**).

Point 3: (Table of strains)

Adding a table of strains seems a very good idea to me indeed, and I have done so (**line 381**, text calls: **line 335, 385**). I must say that I do not really know why there is such a difference in transformation efficiency between Cal13.1B- and T60. However, I would say that Cal13.1B- is quite similar in that respect to T222+, so I’d argue it is T60 that is the outlier with a very low transformation efficiency. T60 is the most cell wall deficient strain I have ever worked with, and the cells are really quite fragile; my guess would be that this is the reason for my difficulties in transforming it.

The fact that Cal13.1B- is not so dissimilar to T60 when it comes to strains recovered photo-autotrophically may reflect an observation made by colleagues in the lab, that Cal13.1B- appears to have depressed levels of PSII subunit D1 in addition to the loss of *RBCS1,2*. However, while my colleagues are currently preparing a publication on a characterization of this strain, I feel that this is beyond the scope of my article and I have therefore refrained from speculating in that direction in the text.
